
JOURNAL INFORMATION
==============================
NLM Title Abbreviation: ScientificWorldJournal
Journal ID: ScientificWorldJournal
NLM Journal ID: 101131163
Journal ID: TSWJ

The Scientific World Journal

ISSN: 2356-6140
EISSN: 1537-744X
Publisher: Wiley

ARTICLE INFORMATION
==============================
PMCID: PMC5956514
PMID: 29861667
DOI: 10.1100/tsw.2004.173
Article ID: 308723
Article version: 1
Subjects: Preface

Biological Effects of Electromagnetic Radiation-Special Issue

Tzaphlidou Margaret 1
Somosy Zoltán 2
1 Medical Physics Laboratory, Medical School, Ioannina University, 45110 Ioannina, Greece
2 “Fodor Jozsef” National Center of Public Health, “Frederic Joliot-Curie” National Research Institute for Radiobiology and Radiohygiene, Department of Radiopathology, Budapest, Hungary
Publication date: 2004
Electronic publication date: 2004 Oct 20
Volume: 4
First page: 1
Last page: 3
Copyright: Copyright © 2004 Margaret Tzaphlidou and Zoltan Somosy.
Copyright year: 2004
License: This is an open access article distributed under the Creative Commons Attribution License, which permits unrestricted use, distribution, and reproduction in any medium, provided the original work is properly cited.
License URL: https://creativecommons.org/licenses/by/3.0/

==============================
Preface
Biological Effects of Electromagnetic Radiation
*TheScientificWorldJOURNAL* (2004) 4(S2), 1–3
ISSN 1537-744X; DOI 10.1100/tsw.2004.173

### Biological Effects of Electromagnetic Radiation

### Special Issue

Margaret Tzaphlidou, Editor[1] and Zoltán Somosy, Assistant Editor[2]

*[1]Medical Physics Laboratory, Medical School, Ioannina University, 45110 Ioannina, Greece, Tel: +30 2651 097595/Fax: +30 2651 097854; [2]"Fodor Jozsef" National Center of Public Health, "Frederic Joliot-Curie" National Research Institute for Radiobiology and Radiohygiene, Department of Radiopathology, Budapest, Hungary, Tel: +36 1 482 2000/ Fax: +36 1 482 2020*

**E-mail:** mtzaphli@cc.uoi.gr, somosy@hp.osski.hu

*Published October 20, 2004*

---

Radiation, ionizing and nonionizing, has always been present all over the earth.

Nonionizing, electromagnetic radiation does not ionize molecules and atoms and, therefore, until a few decades ago, this radiation was not considered particularly dangerous for living organisms. Nevertheless, in the past decades, relatively strong evidence has suggested that nonionizing radiation can be bioactive on cells and biomolecules. Increasing amounts of reliable experimental data and theoretical work now indicate that nonionizing radiation can induce nonthermal effects on living systems. The question — whether there is a link between electromagnetic field exposures and the different biological endpoints of cancer — is of special interest.

During the last 2 decades, a new gadget — the mobile phone — has been introduced into our daily lives. The dramatic increase in mobile phone users during the last 5 years has raised increased concern over the possible side effects of nonionizing radiation.

The biological effects of electromagnetic radiation are a subject that seriously concerns not only the scientific community, but the public too, as more and more evidence has been accumulated about biointeractions that might be harmful to human health. To estimate the potential health risks, we need to answer the conundrum: What is the fundamental mechanism by which electromagnetic fields influence biosystems? To obtain the knowledge of the underlying molecular mechanisms, there is still need for much more basic research in this area.

This special issue of TheScientificWorldJOURNAL intends to present today's international work on the subject, to give particular answers and solutions, and provide the bases for further investigation.

We sincerely thank all the authors for their contribution to this special issue.

©2004 with author.

### TABLE OF CONTENTS

**Preface: Biological Effects of Electromagnetic Radiation**
Margaret Tzaphlidou and Zoltan Somosy

### Review Articles

**Induction of Cell Activation Processes by Low Frequency Electromagnetic Fields**
Myrtill Simkó

**Electromagnetic Fields and Human Endocrine System**
Michal Karasek and Marta Woldanska-Okonska

### Research Articles

**Effects of 2.45-GHz Electromagnetic Fields with a Wide Range of SARs on Micronucleus Formation in CHO-K1 Cells**
S. Koyama, Y. Isozumi, Y. Suzuki, M. Taki , and J. Miyakoshi

**Influence of 50-Hz Electromagnetic Field on Anurian (*Xenopus laevis*) Metamorphosis**
S. Grimaldi, A. Lisi, S. Rieti, V. Manni, M. Ledda, and L. Giuliani

**Effect of 910-MHz Electromagnetic Field on Rat Bone Marrow**
Georgia Demsia, Dimitris Vlastos, and Demetrios P. Matthopoulos

**The Biological Effect of Extremely Low Frequency Electromagnetic Fields and Vibrations on Barley Seed Hydration and Germination**
Armine Amyan and Sinerik Ayrapetyan

**The Effects of 910-MHz Electromagnetic Field on Rat Cranial Arachnoid and Dura Mater Collagen. The Axial Periodicity of Collagen Fibrils**
Margaret Tzaphlidou and Evangelos Fotiou

**Alteration of Tight and Adherens Junctions on 50-Hz Magnetic Field Exposure in Madin Darby Canine Kidney (MDCK) Cells**
Zoltán Somosy, Zsolt Forgács, Gabriella Bognár, Katalin Horváth, and Győző Horváth

**Effects of Whole Body 50-Hz Magnetic Field Exposure on Mouse Leydig Cells**
Zsolt Forgács, Zoltán Somosy, Györgyi Kubinyi, Hanna Sinay, József Bakos, György Thuróczy, András Surján, Aranka Hudák, Ferenc Olajos, and Péter Lázár

**The Effect on Rat Thymocytes of the Simulteneous *in vivo* Exposure to 50-Hz Electric and Magnetic Field and to Continuous Light**
Daniela Quaglino, Miriam Capri, Luigi Zecca, Claudio Franceschi, and Ivonne Pasquali Ronchetti

### Protocol Article

**Development of a Reliable and Low-Cost System for the Study of EMF Biological Effects**
M. Tzaphlidou, E. Fotiou, Ch. Gousias, and D.P. Matthopoulos

**This article should be referenced as follows:**

Tzaphlidou, M. and Somosy, Z. (2004) Biological effects of electromagnetic radiation. *TheScientificWorldJOURNAL* **4(S2)**, 1-3.
